# Electrospinning Synthesis of Na_0.5_Bi_0.5_TiO_3_ Nanofibers for Dielectric Capacitors in Energy Storage Application

**DOI:** 10.3390/nano12060906

**Published:** 2022-03-09

**Authors:** Yuan Liu, Hang Luo, Zhe Gao, Haoran Xie, Ru Guo, Fan Wang, Xuefan Zhou, Jun Cao, Dou Zhang

**Affiliations:** 1 State Key Laboratory of Powder Metallurgy, Central South University, Changsha 410083, China; yuanliu1208@csu.edu.cn (Y.L.); gzgz290036@163.com (Z.G.); xiehr123@csu.edu.cn (H.X.); csuguoru@csu.edu.cn (R.G.); 203312127@csu.edu.cn (F.W.); zhouxuefan@csu.edu.cn (X.Z.); dzhang@csu.edu.cn (D.Z.); 2 Technology Center of China Tobacco Hunan Industrial Co., Ltd., Changsha 410007, China

**Keywords:** electrospinning, Na_0.5_Bi_0.5_TiO_3_ nanofibers, energy density, dielectric composites, capacitors

## Abstract

Dielectric composites based on ferroelectric ceramics nanofibers are attracting increasing attention in capacitor application. In this work, the sol–gel method and electrospinning technology are utilized to prepare one-dimensional Na_0.5_Bi_0.5_TiO_3_ (NBT) nanofibers, and the influence of electrospinning process parameters such as spinning voltage, liquid supply rate, and collector speed on the morphology and structure of nanofibers are systematically explored. The final optimized parameters include the applied voltage of 20 kV, the solution flow rate of 1 mL/h, and the collector’s rotation speed of 1500 rpm. The optimized NBT nanofibers are introduced into the PVDF polymer matrix for energy storage application. Owing to the enhanced interfacial polarization between PVDF matrix and NBT nanofibers with a high aspect ratio, the NBT–PVDF nanocomposites achieve a high discharge energy density of 14.59 J cm^−3^ and an energy efficiency of 53.69% at 490 kV mm^−1^, which are higher than those of pure PVDF, i.e., 10.26 J cm^−3^ and 48.17% at 420 kV mm^−1^, respectively. The results demonstrate that the strategy of synthesizing NBT nanofibers using the electrospinning method is of great potential for high-performance dielectric capacitor application.

## 1. Introduction

Capacitors have attracted much attention for their diverse applications in military and civilian fields, such as pulsed power equipment and flexible direct power transmission, etc., because of the advantages of high-power density and a long service life [1,2,3,4,5]. However, the energy storage density of a capacitor is low, which limits its application in a wider range of fields [6,7]. Dielectric composites composed of ceramic fillers and polymer matrix have the characteristics of high dielectric constant, high breakdown strength, and flexibility. In recent years, some researchers have been paying attention to the exploitation of composites for a dielectric capacitor with high energy storage density and energy efficiency [8,9,10,11,12,13,14].

Studies have found that the morphologies of the ceramic fillers will significantly affect the energy storage performance of the dielectric composites. For example, the surface activity of spherical nanoparticles is large and is easy to agglomerate in the polymer matrix. In this case, defects such as pores will appear at the interface between fillers and matrix, leading to weakened interface polarization, which will result in suppressed dielectric constant and increased dielectric loss in the composites [15,16]. Compared with the nanoparticles, the paralleled two-dimensional nanosheets perform as a number of microcapacitors inside composites. The nanosheets align perpendicular to the electric field, which can be regarded as barriers to block the electrical treeing path [17]. However, it is difficult to synthesize two-dimensional nanosheets. By contrast, the ceramics with one-dimensional structures, such as nanofibers with a high aspect ratio, can not only effectively increase the dielectric constant of the composites but also help to improve the mechanical properties. Fillers with a high aspect ratio in the composites reach the percolation threshold easier and are allowed to communicate or pass continuously in the composites so that the composites prepared with a high aspect ratio will have a higher dielectric constant at a lower loading of fillers [18,19,20]. Compared with ceramic nanofibers with a low aspect ratio, the lower surface area of fillers with a high aspect ratio helps to reduce the surface energy and prevents fillers from agglomerating in the composites. In addition, the ceramic nanofibers with a high aspect ratio possess a large dipole moment of fillers, which enables composites to achieve a higher dielectric enhancement under lower loading [21,22].

Another advantage, one-dimensional fillers with a high aspect ratio can also improve the breakdown strength of composites. The breakdown phase tends to grow on the fragile nanofiber–matrix interface and then penetrate or bypass the fillers near the breakdown path. When the electric field is applied in the out-plane direction of composites, the nanofibers along the in-plane direction exhibit anisotropy, and the breakdown path of the nanofiber composites is straight. When the front end of the breakdown phase encounters the nanofibers, it tends to penetrate the fiber instead of bypassing them. The front edge of the breakdown phase cannot penetrate the nanofibers before the applied electric field reaches a critical value. Therefore, the nanofiber composites can withstand a higher breakdown field than that of the composites with nanoparticle fillers [23,24,25,26]. In summary, composites with high aspect ratio nanofibers are beneficial for improving both the dielectric constant and breakdown strength, which are suitable for dielectric energy storage applications.

Currently, one-dimensional nanofibers can be prepared by electrospinning, template method, hydrothermal/solvothermal, sol–gel, chemical vapor deposition, and physical vapor deposition. In our previous works, the Na_0.5_Bi_0.5_TiO_3_ (NBT) nanowires with a high aspect ratio were synthesized by hydrothermal method and dispersed in the P(VDF–HFP) polymer matrix, resulting in the improved energy storage density of 12.7 J/cm^3^ [27]. Afterwards, Yang et al. synthesized NBT whiskers by a two-step molten salt process and designed sandwich-structured PVDF-based composite film, with a characteristic of the inner PVDF layer filled with NBT whiskers and the outer layers of pure PVDF [28]. The main challenges for these methods include a complex synthesis process and low yield. Electrospinning technology can overcome these shortcomings and continuously prepare nanofibers with a high aspect ratio. This method has the advantages of mild preparation conditions, low equipment requirements, wide application range, simple operation process, and high production efficiency. At present, academia and industry generally believe that electrospinning technology is the most promising process for the industrialization of nanofibers in the 21st century [29,30,31,32,33].

Synthesis of ceramic fillers by electrospinning method mainly includes three steps. Firstly, the electrospinning precursor was prepared by adding polymer to the metal salt sol or inorganic alkoxide and adjusting the viscosity. Then, the precursor fibers were obtained by electrospinning the precursor. Finally, ceramic nanofibers were obtained by sintering the precursor fibers. The basic process of solution electrospinning includes the solution charging under the combined action of electric field force, surface tension and viscous resistance, Taylor cone generation, solution jetting, and then solidification and collection [34,35]. The morphology and microstructure of electrospinning nanofibers are mainly affected by the electrospinning process and sintering process. The electrospinning process is affected by a variety of parameters, such as ambient parameters, solution parameters, and processing parameters. Ambient parameters mainly include temperature and humidity, solution parameters mainly include solution concentration, viscosity, conductivity, and surface tension, and the processing parameters on electrospinning mainly include applied voltage, solution flow rate, and collector’s rotation speed [29,36,37,38]. Indeed, electrospinning technology was previously used in preparing ceramic nanofibers for energy storage applications [39]. However, in previous research reports, there is a lack of research on the process parameters of electrospinning nanofibers, especially for NBT nanofibers. The processing parameters on electrospinning will directly affect the quality of nanofibers.

NBT ceramics have excellent ferroelectric properties and a high dielectric constant, with a maximum polarization strength of about 40 μC cm^−2^ [40,41,42]. However, the current research on the specific effects of the electrospinning parameters for NBT nanofibers is still insufficient, and it is necessary to continue to move forward. In this work, the sol–gel method and electrospinning method were used to prepare NBT nanofibers. The influence of spinning applied voltage, solution flow rate, and collector’s rotation speed on the morphology and structure of NBT nanofibers were explored to obtain the optimal processing parameters. By the controlled variable method, one parameter is changed each time to optimize one by one. The objective is to obtain NBT nanofibers with a nanoscale diameter and high aspect ratio. In this case, fibers are easier to disperse in the PVDF matrix and will form more interfacial regions, both of which are beneficial for better energy storage performance and were proved in previous work. For example, Hu et al. obtained three different diameter distributions of 500–600 nm, 300–400 nm, and around 100 nm by electrospinning, respectively. The nanocomposite films of PZT nanofibers/P(VDF-HFP) contained with different diameter nanofibers were prepared. The dielectric properties of the nanocomposites were comparatively investigated, and the one with the minimum diameter nanofibers exhibited the best performances of 12.58 J cm^−3^ at 470 kV mm^−^^1^ [43]. However, in order to ensure the NBT nanofibers in the composite maintain a high aspect ratio, it is necessary to utilize the NBT nanofibers with better mechanical strength. Therefore, NBT nanofibers with smaller diameters and better strength are generally optimal for the nanocomposites. Subsequently, NBT nanofibers were used to prepare NBT–PVDF nanocomposites with different loadings, and it is a preliminary study that uses electrospinning nanofibers of Na_0.5_Bi_0.5_TiO_3_ in PVDF to get a capacitor. The breakdown strength increased from 420 kV mm^−1^ of pure PVDF to 490 kV mm^−1^ of the nanocomposites with 2 wt% NBT nanofibers. The discharge energy density and energy efficiency were 14.59 J cm^−3^ and 53.69%, respectively, which were higher than those of pure PVDF (10.26 J cm^−3^ and 48.17% at 420 kV mm^−1^, respectively). The improved energy storage performance was a result of the enhanced interfacial polarization. This study provides guidance for the preparation of nanofibers by electrospinning and contributes to a better understanding of improving the energy density and energy efficiency of capacitors.

## 2. Materials and Methods

*Materials:* The raw materials included bismuth acetate (C_6_H_9_BiO_6_, 99.9%, Macklin, Shanghai, China), anhydrous sodium acetate (NaCOOCH_3_, 99.0%, Sinopharm, Shanghai, China), tetrabutyl titanate (Ti(OC_4_H_9_)_4_, 98.0%, Sinopharm, Shanghai, China), acetylacetone (C_5_H_8_O_2_, 99.0%, Sinopharm, Shanghai, China), ethylene glycol monomethyl ether (99.5%, Sinopharm, Shanghai, China), acetic acid (C_2_H_4_O_2_, 99.5%, Sinopharm, Shanghai, China), polyvinylpyrrolidone (PVP, Mw 1300000, Macklin, Shanghai, China), absolute ethanol (CH_3_CH_2_OH, 99.7%, Tianjin Fuyu Fine Chemical Co., Ltd., Tianjin, China), *N*,*N*-dimethylformamide (DMF, 99.5%, Sinopharm, Shanghai, China), and PVDF (6020, Solvay, Shanghai, China).

*Preparation of NBT sol for electrospinning:* 4.0542 g of bismuth acetate and 0.8612 g of anhydrous sodium acetate were added into 30 mL of ethylene glycol methyl ether and acetic acid mixed solution. The mixed solution was stirred at 90 °C for 30 min to become a clear solution, which was recorded as liquid A. The volume ratio of ethylene glycol methyl ether and acetic acid was 1:1, and the 5% excess bismuth and sodium acetate were used to compensate for the possible loss of bismuth and sodium during high-temperature annealing. Then, 6.8064 g of tetrabutyl titanate was dissolved in 4.0044 g of acetylacetone to prevent it from being hydrolyzed in the air and stirred at 40 °C for 30 min to obtain an orange solution, which was recorded as liquid B. Liquid B was added into solution A slowly, then an appropriate amount of ethylene glycol methyl ether was added into the constant volume, and stirred at 40 °C for 12 h to obtain NBT sol, which was recorded as solution C.

*Preparation of NBT nanofibers by electrospinning:* PVP and alcohol were mixed at a mass ratio of 1:4 and stirred at room temperature for 24 h to obtain a PVP alcohol solution, which was recorded as liquid D. Liquid C and liquid D were mixed in different proportions and stirred at 40 °C for 24 h to form a uniform solution. The mixed solution was put into a 10 mL disposable plastic syringe with a needle for spinning. The temperature of the electrospinning ambient was controlled at 40–50 °C, the humidity was lower than 20%, and the tip to collector distance was 15 cm. The precursor nanofibers were collected and dried at 60 °C for 48 h, and then kept at 325 °C and 700 °C for 1 h to obtain high aspect ratio NBT nanofibers.

*Fabrication of the NBT–PVDF nanocomposites:* The NBT nanofibers were ultrasonically dispersed in a DMF solution for 30 min. Then, PVDF powders were added into the mixture solution in proportion and stirred at 40 °C for 24 h. The NBT–PVDF suspensions with different weight fractions (2 wt%, 4 wt%, 6 wt%, 8 wt%) were prepared. The suspensions were evenly coated on the glass substrate by solution casting method and dried at 80 °C for 12 h in a vacuum. The nanocomposites were hot pressed at 210 °C for 30 min followed by immediately quenching in ice water, and then vacuum dried at 80 °C for 12 h to volatilize the residual water.

*Characterization:* The microstructures of electrospinning NBT nanofibers were characterized with XRD (Advance D8, Bruker company, Billerica, MA, USA), SEM (MIRA4 LMH Ultim Max 40, Tescan company, Brno, Czech), HRTEM (Titan G2 60-300, FEI Company, Hillsboro, OR, USA), and TGA (TGA/STA 8000-FTIR-GCMS, PerkinElmer, Fremont, CA, USA). The dielectric properties of NBT–PVDF nanocomposites were characterized with Agilent 4990A (Palo Alto, Santa Clara, CA, USA). The electric displacement hysteresis loops were obtained from the ferroelectric test system (Aix ACCT, TF-2000, Aachen, Germany) at 10 Hz. The thickness of the samples can be controlled by adjusting the height of the stainless steel scraper. The precision of the stainless steel scraper is 1 μm. The electric field applied to each sample is gradually increased until the breakdown to obtain the breakdown electric field value. The number of test samples is 20. The average thickness of each sample ranges from 10 to 15 μm due to the different loading amounts of NBT nanofibers and the difference of surface roughness of glass substrate. All of the samples are obtained from the solution after the solvent is volatilized. The cross section of nanocomposites was obtained after brittle fracture with liquid nitrogen.

## 3. Results and Discussion

**Figure 1:**
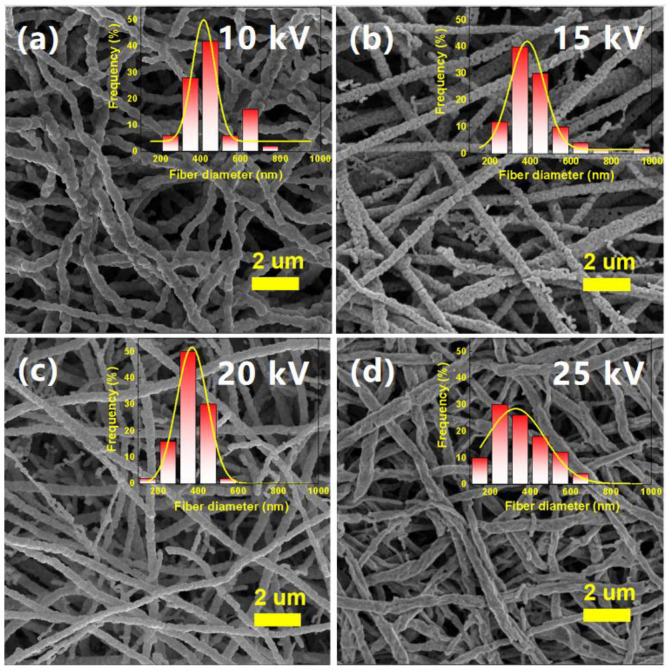
SEM images of NBT nanofibers obtained under different applied voltages at the solution flow rate of 1.0 mL/h and collector’s rotation speed of 1500 rpm: (**a**) 10 kV; (**b**) 15 kV; (**c**) 20 kV; (**d**) 25 kV. The inserted figures are the diameter distributions of NBT nanofibers.

There is a certain range requirement for the applied voltage on electrospinning, and only when the applied voltage reaches the threshold voltage will the jet be formed, and if the voltage is too high, the jet will be broken down [44,45]. The solution flow rate of 1.0 mL/h and collector’s rotation speed of 1500 rpm were fixed, and only the applied voltage was controlled. The morphology of the samples obtained after heat treatment is shown in Figure 1. The average fiber diameter decreases from 446 nm at 10 kV to 350 nm at 25 kV with the increase of the applied voltage (Table 1). This is because the electrostatic repulsion of the fluid jet will be increased with the increasing of the applied voltage during the electrospinning process, which is conducive to the reduction of fiber diameter. The high applied voltage can increase the coulomb force carried by the droplet, accelerating the jet stretching and causing the fiber diameter to decrease. Figure 1a shows that the sample at 10 kV is not well stretched; the nanofibers are slightly curved and connected together, which is not conducive to subsequent dispersion. The jet flow at 25 kV is highly unstable. In this case, the nanofibers cannot be well collected on the collector. Most of the nanofibers will find a closer grounding device to deposit, the output of nanofibers will decrease significantly, and the high applied voltage will increase the risk of experiments. The average diameter of the fibers is 360 nm at 20 kV, and the linearity of nanofibers is great. Considering comprehensively, the applied voltage of 20 kV is selected.

**Figure 2:**
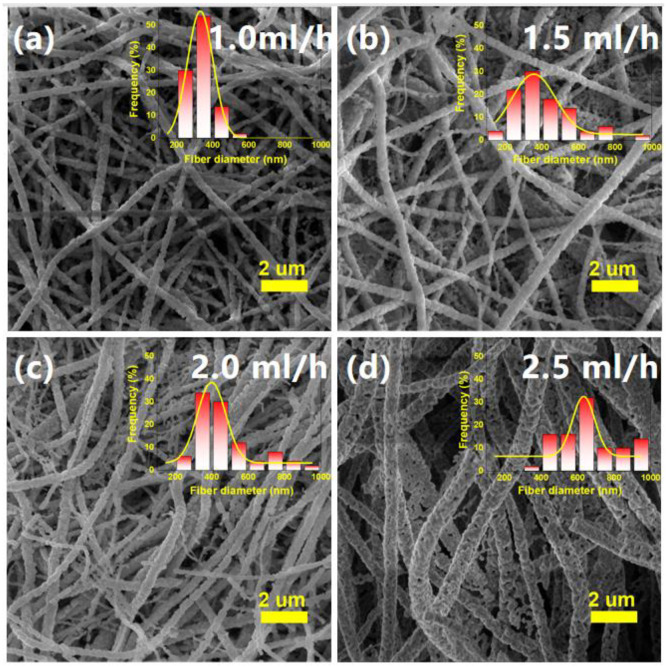
SEM images of NBT nanofibers obtained under different solution flow rates at the applied voltage of 20 kV and collection’s rotation speed of 1500 rpm: (**a**) 1.0 mL/h; (**b**) 1.5 mL/h; (**c**) 2.0 mL/h; (**d**) 2.5 mL/h. The inserted figures are the diameter distributions of NBT nanofibers.

The solution flow rate in the syringe has a very important influence on the fiber conversion rate on solution electrospinning. In this experiment, the solution flow rate is controlled with the applied voltage of 20 kV and the collection’s rotation speed of 1500 rpm. As shown in Figure 2, the diameter of NBT nanofibers is increased from 326 nm at 1.0 mL/h to 660 nm at 2.5 mL/h with the increase in the solution flow rate and the uneven distribution of fibers became more and more serious. This is because the solution jet rate becomes slower, and the solvent has sufficient time to volatilize at a lower solution flow rate, which is more beneficial to fiber formation [46,47]; with a higher solution flow rate, the polymer segments per unit of time are more aggregated. The chain segment is not fully stretched at the same time, and the formed fiber melts, and the diameters of fibers and pores increase accordingly (Figure 2d). However, if the solution flow rate is too low, it can also cause discontinuity of the solution flow. Therefore, controlling a certain range of solution flow rate has an important influence on the morphology of the spun nanofibers. As shown in Figure 2a, when the solution flow rate is 1.0 mL/h, the nanofibers have a good morphology, and the average diameter is 326 nm. Therefore, the recommended solution flow rate is 1.0 mL/h.

**Figure 3:**
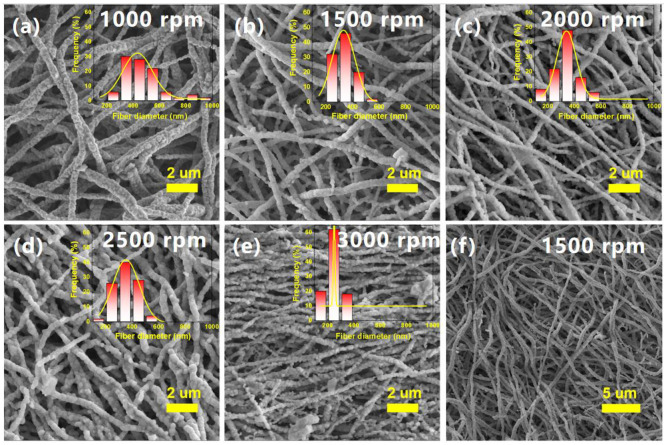
SEM images of NBT nanofibers obtained under different collector rotation speeds at the applied voltage of 20 kV and solution flow rate of 1.0 mL/h: (**a**) 1000 rpm; (**b**,**f**) 1500 rpm; (**c**) 2000 rpm; (**d**) 2500 rpm; (**e**) 3000 rpm. The inserted figures are the diameter distributions of NBT nanofibers.

The diameter of the fiber can be well regulated by changing the rotation speed of the drum collector. The influence of the rotating speeds of the drum collector on the morphology of NBT nanofibers at the applied voltage of 20 kV and solution flow rate of 1.0 mL/h was explored. As shown in Figure 3, the diameter of nanofibers is decreased from 467 nm at 1000 rpm to 242 nm at 3000 rpm. It means that with the increasing of collector’s rotation speed, the nanofibers gradually display from random arrangement to orientation characteristics. The diameter and morphology of the NBT nanofibers obtained at 1500 rpm are greater than others, and 46% of NBT nanofibers’ diameters are in the range of 300–400 nm (Figure 3a,f). When the collector’s rotation speed is 2000 rpm, the fiber diameter distribution is widened. This is because the collector’s rotation speed is fast, and some fibers are broken during the electrospinning processing. When the collector’s rotation speed is 2500 rpm, the fiber diameter increases slightly. When the collector’s rotation speed reaches 3000 rpm, the NBT nanofibers show overall orientation characteristics, the range of diameters is 150–360 nm. It is considered that the diameter of the nanofibers is too small, which will seriously affect the mechanical strength and is not conducive to subsequent experiments. In addition, if the collector’s rotation speed is too high, the drum collector produces a lot of heat, which affects the safety of the experiment and the service life of the electrospinning device. The recommended rotation speed of the collector is 1500 rpm.

**Figure 4:**
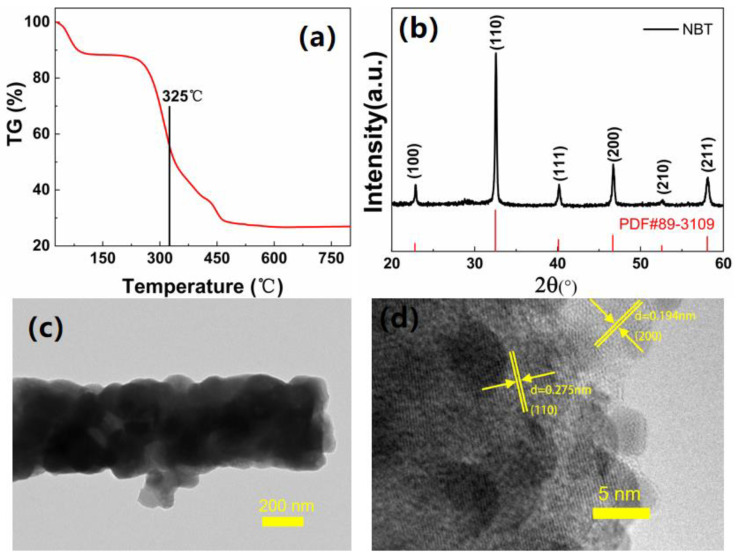
(**a**) TGA curve of electrospinning nanofiber; (**b**) XRD pattern; (**c**) TEM; (**d**) HRTEM of NBT nanofibers.

To understand the calcination process, the thermogravimetric analysis (TGA) was conducted (Figure 4a). In the TGA curve, the weight losses are 11.64% (25–200 °C), 51.7% (200–400 °C), and 9.32% (400–750 °C) originating from the solvent vaporization, acetate ligand decomposition, and pyrolysis of gel, respectively [48,49]. According to the TGA test result, the electrospinning nanofibers were incubated at 325 °C and 750 °C for 1 h, respectively, and finally, NBT nanofibers were obtained. The XRD patterns of nanofibers present the typical NBT data (Figure 4b). The microstructure of the NBT nanofibers was further analyzed by TEM, as shown in Figure 4c. The HRTEM image in Figure 4d illustrates the crystalline interplanar spacings of 0.275 nm and 0.194 nm, corresponding to the (110) and (200) planes of the NBT crystal.

**Figure 5:**
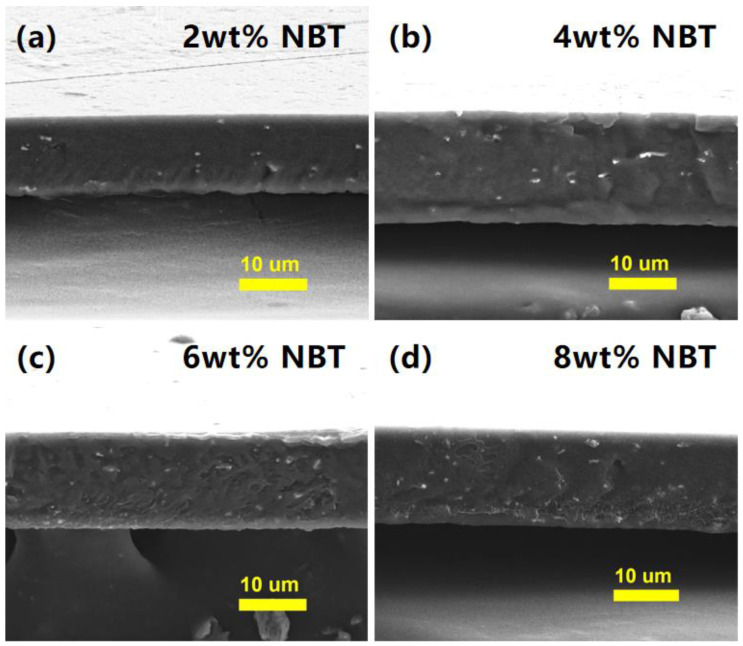
Cross-sectional SEM images of the NBT–PVDF nanocomposites. (**a**) 2 wt% NBT, (**b**) 4 wt% NBT, (**c**) 6 wt% NBT and (**d**) 8 wt% NBT.

Optimized parameters of applied voltage, solution flow rate, and collector’s rotation speed are 20 kV, 1.0 mL/h, and 1500 rpm, respectively. In these conditions, the synthesized NBT nanofibers were utilized for dielectric energy storage applications, and NBT nanofibers were introduced into PVDF to prepare nanocomposites. The cross section SEM images of the NBT–PVDF with different loading of NBT nanofibers are shown in Figure 5. NBT nanofibers with a high aspect ratio showing no obvious aggregation are observed in the PVDF matrix. The film surface is smooth and flat, which helps obtain excellent dielectric energy storage performance.

**Figure 6:**
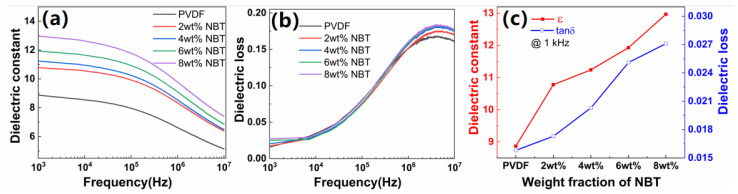
The frequency-dependent changes of (**a**) dielectric constant and (**b**) dielectric loss of NBT–PVDF nanocomposites; (**c**) dielectric constant and dielectric loss of NBT–PVDF nanocomposites with various loadings of NBT nanofibers at 1 kHz.

The dielectric properties of the NBT–PVDF nanocomposites were investigated. Figure 6a shows the dielectric constant of the NBT–PVDF nanocomposites with frequencies. Due to the dielectric relaxation of PVDF, the dielectric constant of the NBT–PVDF nanocomposites decreases with the increase in frequency. For example, the dielectric constant of 8 wt% NBT–PVDF is 12.97 at 1 kHz and 7.41 at 10 MHz, respectively. Figure 6b displays dielectric loss of the nanocomposites increases with an increase in frequency from 1 kHz to 10 MHz, which is mainly related to interface polarization and molecular dipole motion. The dielectric constant of the nanocomposites increases significantly with the increase in the NBT nanofibers loading. Compared with the 8.87 pure PVDF at 1 kHz, the dielectric constants of 2 wt%, 4 wt%, 6 wt% and 8 wt% NBT–PVDF nanocomposites achieved increases of 21.5%, 26.7%, 34.5%, and 46.2%, respectively (Figure 6c). This is mainly attributed to the following reasons. Firstly, the NBT nanofibers with a high dielectric constant are introduced into the PVDF matrix, and the dielectric constant of the nanocomposites increases with the NBT nanofibers loading. Secondly, a major role was attributed to the interphase and the interaction zone between the NBT nanowires and the PVDF matrix. For ferroelectric nanocomposite, the interphase of the dielectric permittivity is higher than that of the matrix because of the polarization enhancement in the interphase [4,50,51,52]. As the loading of NBT nanofibers increases, it is inevitable to introduce a small number of defects. For instance, dielectric loss increases from 0.0158 of pure PVDF to 0.0271 of 8 wt% NBT–PVDF nanocomposite at 1 kHz.

**Figure 7:**
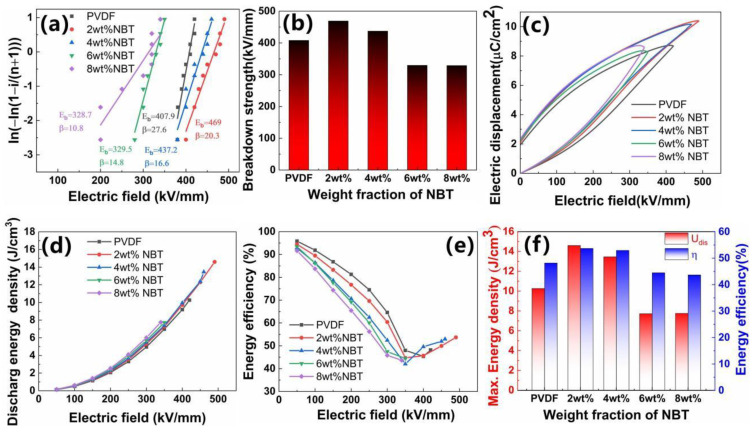
(**a**) Weibull distribution, (**b**) characteristic breakdown strength, (**c**) displacement hysteresis loops, (**d**) *U*_dis_, (**e**) η, and (**f**) *U*_dis_ and η at each *E_b_* of NBT–PVDF nanocomposites.

Figure 7 shows the energy storage performance of NBT–PVDF nanocomposites. The Weibull distribution function is described as the following equation [53]:

(1) $$P(E)=1-\exp(-{\left(E/E_{b}\right)}^{\beta })$$

where *E* represents the experimental breakdown electric field, and *E_b_* represents the characteristic electric breakdown strength from the Weibull distribution. The parameter *β* is the slope of the line after linear fitting. Figure 7a,b display Weibull distribution and *E_b_* of NBT–PVDF nanocomposites. It is found that with the increase in NBT nanofibers loading, the *E_b_* value increases firstly and then decreases. For instance, the *E_b_* and *β* of pure PVDF, 2 wt%, 4 wt%, 6 wt%, and 8 wt% NBT–PVDF nanocomposites are 407.9 kV mm^−1^ (*β*~27.6), 469.0 kV mm^−1^ (*β*~20.3), 437.2 kV mm^−1^ (*β*~16.6), 329.5 kV mm^−1^ (*β*~14.8), and 328.7 kV mm^−1^ (*β*~10.8), respectively. The NBT nanowires with a high aspect ratio are trended to arrange in the direction perpendicular to the electric field during the casting process because of gravity, which can limit the charge migration. The electrical tree tends to expand on the fragile nanofiber–matrix interface. When the front end of the breakdown phase encounters the nanofiber, it tends to penetrate the fiber instead of bypassing it [24,54]. Therefore, the composites with the optimized amount of NBT nanofibers can withstand a higher breakdown field. When the loading of NBT nanofibers is further increased to 4 wt%, the excessive filling of NBT nanofibers causes severe distortion of the local electric field and structural defects in the interface area, resulting in the decreased breakdown electric field. Figure 7c shows the typical displacement hysteresis loops of NBT–PVDF nanocomposites. The 2 wt% NBT–PVDF composite has a maximum electric displacement of 10.39 μC cm^−2^ and a small residual polarization of 2.091 μC cm^−2^ at 490 kV mm^−1^. The *U*_dis_ can be calculated according to the formula $U_{\mathrm{dis}}=\int EdD$, where E and *D* are electric breakdown strength and electric displacement, respectively [5,55]. The curve of the discharge energy density (*U*_dis_) and energy efficiency (η) of nanocomposites with electric field is shown in Figure 7d,e. It can be seen from Figure 7d,e the *U*_dis_ increases with the increase in the electric field. The η decreases first and then increases with the increase in the electric field due to the relaxation phenomenon of PVDF. The *U*_dis_ and η of pure PVDF, 2 wt%, 4 wt%, 6 wt% and 8 wt% NBT–PVDF nanocomposites are 10.26 J cm^−3^ (48.17%), 14.59 J cm^−3^ (53.69%), 13.46 J cm^−3^ (52.94%), 7.72 cm^−3^ (44.49%) and 7.75 cm^−3^ (43.67%), respectively. It is worth noting in Figure 7f the highest *U*_dis_ of 14.59 Jcm^−3^ is obtained in 2 wt% NBT–PVDF composites at 490 kV mm^−1^, which is 1.42 times that of pure PVDF. At the same time, the nanocomposites achieve the highest η of 53.7%, which is ~5.5% higher than that of pure PVDF (η~48.17% at 420 kV mm^−1^). Some related results have been added in Table 2 to make the comparison [18,27,56,57,58]. Besides dielectric constants, we also focus on the breakdown field strength and energy storage density for comparison. It is found that our breakdown electric field and energy storage density are the highest, as shown in Table 2.

## 4. Conclusions

NBT nanofibers with a high aspect ratio and a uniform diameter distribution were synthesized by electrospinning. The parameters of applied voltage, solution flow rate, and collector rotation speed in the electrospinning process were optimized as 20 kV, 1.0 mL/h, and 1500 rpm, respectively. Subsequently, the optimized NBT nanofibers were introduced into the PVDF matrix to prepare dielectric nanocomposites for energy storage applications. The NBT nanofibers with a high aspect ratio are easily dispersed into the PVDF matrix uniformly. The NBT–PVDF nanocomposites achieved increased dielectric constant and maintained the advantage of a high breakdown electric field. The results showed that the NBT–PVDF nanocomposites with 2 wt% NBT nanofibers loading achieved a higher discharge energy density of 14.59 J cm^−3^ and energy efficiency of 53.69% owing to the enhanced interfacial polarization. This study provided guidance for preparing NBT nanofibers by the electrospinning method and proved that high aspect ratio NBT nanofibers can effectively improve the energy storage performance of PVDF nanocomposites.

## Figures and Tables

**Table 1:** NBT nanofibers diameters under different parameters and the relative contribution of each parameter.

Variable	Applied Voltage (kV)				Solution Flow Rate (mL/h)				Collector Rotation Speed (rpm)				
Set values	10	15	20	25	1.0	1.5	2.0	2.5	1000	1500	2000	2500	3000
d_min_ (nm)	260	210	180	100	200	180	200	310	220	200	150	180	150
d_max_ (nm)	720	1000	510	670	500	960	1000	1000	960	500	500	500	360
d_average_ (nm)	446	407	360	350	326	412	472	660	467	336	331	344	242
Optimized value	20 kV				1.0 mL/h				1500 rpm				
Contribution	Straight				Diameter				Orientation				

**Table 2:** Comparison of the dielectric constant (*ε_r_*), breakdown strength (*E_b_*), energy efficiency (η), and discharge energy density (*U*_dis_) for some typical dielectric composites.

Matrix	Fillers/Loadings	*ε_r_*@1 kHz	*E_b_* (kV/mm)	η (%)	*U*_dis_ (J/cm^3^)	Ref.
PVDF	BaTiO_3_ nanofibers/4 vol%	14.69	370	/	8.78	[18]
PVDF	BaTiO_3_@Al_2_O_3_ nanofibers/2.5vol%	~11	380	65.1	7.1	[56]
PVDF	Ba_0.6_Sr_0.4_TiO_3_ nanofibers-APS/2.5 vol%	~12	380	60	6.8	[57].
P(VDF–CTFE)	Dopa@BaTiO_3_ nanowires/3 vol%	~10.5	354.9	61.4	10.8	[58]
P(VDF–HFP)	Dopa@Na_0.5_Bi_0.5_TiO_3_ nanofibers/2.37vol%	~13	458	/	12.7	[27]
PVDF	Na_0.5_B_i0.5_TiO_3_ nanowires/2wt%	10.78	490	53.69	14.59	This work

## Data Availability

The data presented in this study are available from the corresponding author on request.
